# Systemic inflammation and intelligence in early adulthood and subsequent risk of schizophrenia and other non-affective psychoses: a longitudinal cohort and co-relative study

**DOI:** 10.1017/S0033291718000831

**Published:** 2018-04-06

**Authors:** Nils Kappelmann, Golam M Khandaker, Henrik Dal, Jan Stochl, Kyriaki Kosidou, Peter B Jones, Christina Dalman, Håkan Karlsson

**Affiliations:** 1 Department of Psychiatry, University of Cambridge, Cambridge, UK; 2 Department of Psychiatry, University of Oxford, Oxford, UK; 3 Cambridgeshire and Peterborough NHS Foundation Trust, Cambridge, UK; 4 Centre for Epidemiology and Community Medicine, Stockholm County Council, Stockholm, Sweden; 5 Department of Kinanthropology, Charles University, Prague, Czech Republic; 6 Department of Public Health Sciences, Karolinska Institutet, Stockholm, Sweden; 7 Department of Neuroscience, Karolinska Institutet, Stockholm, Sweden

**Keywords:** Cohort study, co-relative analysis, ESR, immunopsychiatry, inflammation, intelligence, IQ, non-affective psychoses, population-based study, schizophrenia

## Abstract

**Background:**

Schizophrenia is associated with impaired neurodevelopment as indexed by lower premorbid IQ. We examined associations between erythrocyte sedimentation rate (ESR), a marker of low-grade systemic inflammation, IQ, and subsequent schizophrenia and other non-affective psychoses (ONAP) to elucidate the role of neurodevelopment and inflammation in the pathogenesis of psychosis.

**Methods:**

Population-based data on ESR and IQ from 638 213 Swedish men assessed during military conscription between 1969 and 1983 were linked to National Hospital Discharge Register for hospitalisation with schizophrenia and ONAP. The associations of ESR with IQ (cross-sectional) and psychoses (longitudinal) were investigated using linear and Cox-regression. The co-relative analysis was used to examine effects of shared familial confounding. We examined mediation and moderation of effect between ESR and IQ on psychosis risk.

**Results:**

Baseline IQ was associated with subsequent risk of schizophrenia (adjusted HR per 1-point increase in IQ = 0.961; 95% confidence interval (CI) 0.960–0.963) and ONAP (adjusted HR = 0.973; 95% CI 0.971–0.975). Higher ESR was associated with lower IQ in a dose-response fashion. High ESR was associated with *increased* risk for schizophrenia (adjusted HR = 1.14; 95% CI 1.01–1.28) and *decreased* risk for ONAP (adjusted HR = 0.85; 95% CI 0.74–0.96), although these effects were specific to one ESR band (7–10 mm/hr). Familial confounding explained ESR-IQ but not ESR-psychoses associations. IQ partly mediated the ESR-psychosis relationships.

**Conclusions:**

Lower IQ is associated with low-grade systemic inflammation and with an increased risk of schizophrenia and ONAP in adulthood. Low-grade inflammation may influence schizophrenia risk by affecting neurodevelopment. Future studies should explore the differential effects of inflammation on different types of psychosis.

## Introduction

The neurodevelopmental hypothesis of schizophrenia posits that abnormal neurodevelopment contributes to the pathogenesis of the illness (Murray & Lewis, 1987; Weinberger, 1987). This hypothesis is supported by population-based longitudinal studies showing an association of schizophrenia and other non-affective psychoses (ONAP) with lower IQ during childhood/premorbid period (Jones *et al.* 1994; Crow *et al.* 1995; David *et al.* 1997; Davidson *et al.* 1999; Cannon *et al.* 2000, 2002; Zammit *et al.* 2004; Reichenberg *et al.* 2005; Kendler *et al.* 2015). These findings have been summarised in a meta-analysis, which shows that there is a linear association between premorbid IQ and risk of adult schizophrenia and related psychoses; for every one-point decrease in IQ risk for schizophrenia increases by 3.7% (Khandaker *et al.* 2011).

Co-relative control analysis is a way to examine familial confounding. This approach compares effect sizes for a particular risk factor-disease association between the general population and groups of relatives with different levels of relatedness (i.e. full siblings, half-siblings, cousins). If the effect size decreases in groups of relatives, compared with general population, this indicates that the association may be explained by shared familial confounding. Using co-relative control analysis, Kendler and colleagues reported that lower cognitive functioning in schizophrenia and ONAP is unlikely to be explained by familial confounding. Furthermore, premorbid cognitive deficits in psychosis may be the result of unique environmental factors (Kendler *et al.* 2015, 2016*a*, *b*). These findings provide important insights into potential causal models of psychosis and warrant exploration of unique environmental factors that may contribute to lower cognitive functioning in psychosis.

Emerging evidence indicates a role for low-grade inflammation in the pathogenesis of psychotic disorders (Khandaker & Dantzer, 2015; Khandaker *et al.* 2015*a*, 2017*a*). Concentrations of circulating inflammatory markers such as interleukin 6 (IL-6) and C-reactive protein (CRP) are elevated during acute psychotic episodes, which tend to normalise after recovery but continue to be elevated in treatment-resistant patients (Potvin *et al.* 2008; Miller *et al.* 2011; Goldsmith *et al.* 2016). Elevated serum concentrations of IL-6 and CRP in childhood/ adolescence are associated with increased risks of psychotic symptoms or diagnosis of schizophrenia subsequently in adulthood (Khandaker *et al.* 2014; Metcalf *et al.* 2017), which indicates that reverse causality is an unlikely explanation for the observed association between low-grade inflammation and psychosis. A functional genetic variant in the *IL-6R* gene known to dampen down inflammation (Ferreira *et al.* 2013) is protective for psychosis and/or severe depression (Khandaker *et al.* 2017*b*) and physical illness commonly comorbid with psychosis such as coronary heart disease (IL6R Genetics Consortium & Emerging Risk Factors Collaboration, 2012) as shown using Mendelian randomisation. This suggests that the inflammation–psychosis relationship is unlikely to be explained by confounding fully.

Despite a great deal of cross-sectional case-control studies linking low-grade inflammation with psychosis (Goldsmith *et al.* 2016), population-based longitudinal studies are relatively rare (Gardner *et al.* 2013; Wium-Andersen *et al.* 2013; Khandaker *et al.* 2014; Metcalf *et al.* 2017). To our knowledge, no longitudinal study has examined the association between erythrocyte sedimentation rate (ESR), a surrogate marker of systemic inflammation and particularly acute phase reaction (Harrison, 2015), and subsequent risk of schizophrenia and related psychosis.

Low-grade inflammation can influence cognitive function across the life-course in the healthy population and in people with schizophrenia. We have reported that elevated CRP is associated with lower IQ in childhood in the ALSPAC birth cohort (MacKinnon *et al.* 2017). We have also reported that there is an inverse relationship between ESR and IQ based on a sample of healthy Swedish adolescents/young-adults (Karlsson *et al.* 2010). Similarly, higher levels of CRP are associated with a lower cognitive function in patients with schizophrenia (Dickerson *et al.* 2007). It is plausible that low-grade inflammation influences psychosis risk by affecting neurodevelopment. However, studies examining whether IQ mediates and/or moderates the association between ESR and subsequent psychotic disorders or whether they are independent risk factors are scarce.

The present study aimed to examine the relationship between inflammation (indexed by ESR), neurodevelopment (indexed by IQ), and subsequent risk of hospitalisation with non-affective psychoses in adulthood in a Swedish population-based sample of 638 213 male conscripts. First, we examine the relationships between lower cognitive functioning at 18–20 years and subsequent diagnosis of psychoses (Jones *et al.* 1994; Zammit *et al.* 2004; Khandaker *et al.* 2011; Kendler *et al.* 2015). Second, we examine the association between inflammation and IQ reported previously from our smaller population-based Swedish sample (Karlsson *et al.* 2010). Third, we test the relationship between inflammation and subsequent diagnosis of psychotic illness. In addition, we examine whether the ESR-psychoses relationship and ESR-IQ association could be explained by shared familial factors using co-relative analysis. We examine mediation and moderation between ESR and IQ with regards to risk of psychoses.

## Materials and methods

### Study population and diagnosis of psychosis

The present study is based on Swedish men conscripted into military between 1969 and 1983. In Sweden, all men underwent obligatory military conscription between ages 18 and 20 years bar certain exceptions, so this representative sample included approximately 97% of the male population (Karlsson *et al.* 2010). The conscripts underwent various assessments over 2 days that tested their suitability for military service. The data provide a unique opportunity for epidemiological investigations; see Ahlborg *et al.* (1973). The conscription data were linked to the Swedish National Hospital Discharge Register until end of 2011, resulting in an average follow-up period of 35 years (range: 21–42 years) after conscription (mean age at follow-up: 54 years; range: 45–68 years). The discharge register contains medical diagnoses defined by the International Classification of Diseases (ICD; World Health Organization, 2004). This allowed the identification of individuals first hospitalised with a discharge diagnosis of schizophrenia (ICD-10: F20; ICD-9 and ICD-8: 295) or ONAP (ICD-10: F21 = Schizotypal Disorder, F22 = Persistent Delusional Disorder, F23 = Acute and Transient Psychotic Disorders, F24 = Induced Delusional Disorder, F25 = Schizoaffective Disorder, F28 = Other Non-organic Psychotic Disorders, F29 = Unspecified Non-organic Psychosis; corresponding diagnostic codes in ICD-9: 297, 298.2-9, 291.3, 291.5, 292.1; ICD-8: 297.0-9, 298.2-3, 298.9, 291.2-3). See online Supplementary Fig. 1 for sample selection process.

### Measurements of IQ and ESR

Intelligence was measured during military conscription by tests on logic/general intelligence, verbal intelligence, visuospatial perception, and mechanical skills in solving mathematical or physical problems; see Karlsson *et al.* (2010). Results of these tests were aggregated on a nine-point scale that approximately corresponds to Wechsler adult IQ bands <74, 74–81, 82–89, 90–95, 96–104, 105–110, 111–118, 119–126, and >126. For visibility and comparison purposes, we rescaled IQ scores from this 9-point scale to a continuous score with mean = 100 (*SD* = 15).

ESR was measured from venous blood samples taken at conscription. ESR is the rate of sedimentation of red blood cells measured over an hour and is a marker of inflammation, particularly a surrogate marker of acute phase reaction (Harrison, 2015). As with our previous Swedish study (Karlsson *et al.* 2010), ESR was corrected for the erythrocyte volume in the blood (i.e. haematocrit), according to the formula ESR × (haematocrit /45), and grouped into four bands, 0–3, 4–6, 7–10, and ⩾11 mm/h. The distribution of ESR was skewed (mean = 3.38; median = 2.18; IQR = 1.87–4.00), so this categorisation allowed better investigation of the effect of high ESR levels on IQ/psychosis. In addition, we carried out sensitivity analyses: (1) by grouping ESR into quartiles; (2) by grouping ESR into deciles; (3) using ESR as a continuous variable; and (4) after excluding participants with very high ESR that may be indicative of an infection; according to Mayo clinic, in men ESR>22 mm/h indicates acute infection.

## Covariates

Migration status (either/both parents born outside Sweden), winter birth (December–May), parental history of schizophrenia and/ or ONAP, household crowding, and maximum parental socioeconomic status at child's age of 8–12 years (worker, white collar professional, business owner, and unknown) were included as covariates.

### Statistical analysis

First, to analyse the association of IQ and ESR with schizophrenia or ONAP, Cox regression was used to estimate hazard ratios (HRs) and 95% confidence intervals (CIs) for each one-point increase in IQ (and separately, for individuals in the higher ESR bands compared with ESR band 0–3 mm/h). Further analyses were carried out adjusting for potential confounders, and after excluding 622 participants hospitalised within 2 years of conscription to minimise reverse causality. Second, we performed co-relative analyses to determine whether HRs for the association between ESR and schizophrenia or ONAP changed as a function of family relatedness. To this end, we applied stratified Cox regression models on respective outcomes in the general population, cousin, half-sibling, and full-sibling pairs. For analyses of relative pairs, robust standard errors were calculated to adjust for family relatedness, that is, we included a cluster effect of each family pair. Furthermore, relative pairs falling into equal ESR bands were excluded to allow identifiability/convergence of the statistical model. We tested the proportionality assumption across all models. We also performed co-relative analyses of the ESR-IQ association. Due to the continuous outcome variable and cross-sectional association, we estimated linear mixed-effects models with outcome IQ and categorical ESR predictors as before (again excluding relative pairs with equal ESR). The models included a random intercept for relative pairs to stratify analyses. Third, we tested the association between ESR (predictor) and IQ (outcome) using linear regression before and after adjusting for potential confounders.

Fourth, we tested whether IQ mediated or moderated the relationship between ESR and schizophrenia or ONAP. For these analyses, we used logistic and linear regression models. We tested mediation by calculating direct and indirect effects of ESR on psychoses. Mediation analysis of the ESR-psychoses relationship by IQ was not conducted for outcome schizophrenia as the unadjusted main analysis did not reach statistical significance, which is a prerequisite for testing mediation. However, mediation analysis for the outcome of ONAP was conducted using the subgroups of individuals with ESR of 0–3 mm/h or 7–10 mm/h, in which main analyses had shown an association between ESR and psychosis. We were also able to run mediation analyses of these ESR groups for both schizophrenia and ONAP when adjusting for covariates. Moderation was tested by including an interaction term (IQxESR) in logistic regression models of schizophrenia and ONAP.

## Results

### Baseline characteristics

Of the total sample of 638 213 Swedish men, at baseline 470 923 (73.79%) had an ESR level of 0–3 mm/h, 112 089 (17.56%) of 4–6 mm/h, 35 023 (5.49%) of 7–10 mm/h, and 20 178 (3.16%) of ⩾11 mm/h. ESR groups differed significantly in age at conscription, migration status, winter birth, parental socioeconomic status, and household crowding (all *p* < 0.05; Table 1). In total, 5398 (0.85%) and 5133 (0.80%) individuals in this population-based sample developed schizophrenia or ONAP until follow-up, respectively.

**Table 1.** Baseline characteristics of the sample

Characteristic	All participants	Groups based on ESR Levels at Conscription in mm/h				
		0–3	4–6	7–10	⩾11	*p* Value^a^
Sample size, N (% of total)	638 213	470 923 (73.79)	112 089 (17.56)	35 023 (5.49)	20 178 (3.16)	−
ESR, *M* (*SD*)	3.38 (3.32)	2.05 (0.85)	5.00 (0.83)	8.45 (1.14)	16.70 (6.94)	<0.001
IQ at conscription, *M* (*SD*)	100.00 (15.00)	100.20 (15.00)	99.71 (14.96)	99.23 (15.04)	98.61 (14.98)	<0.001
Age at conscription, *M* (*SD*)	18.38 (0.75)	18.38 (0.75)	18.37 (0.75)	18.39 (0.74)	18.40 (0.71)	<0.001
Age at end of follow-up, *M* (*SD*)	53.76 (4.26)	53.72 (4.32)	53.73 (4.05)	53.99 (4.11)	54.38 (4.17)	<0.001
Parental history of psychoses, *N* (*%*)	17 161 (2.69)	12 698 (2.70)	2991 (2.67)	923 (2.64)	549 (2.72)	0.863
Migration Status, *N* (*%*)	95 318 (14.94)	71 284 (15.14)	16 174 (14.43)	4940 (14.11)	2920 (14.47)	<0.001
Winter birth, *N* (*%*)	330 651 (51.81)	243 609 (51.73)	58 136 (51.87)	18 295 (52.24)	10 611 (52.59)	0.032
Parental socioeconomic status at age 8–12 years						
Worker, *N* (*%*)	235 705 (36.93)	171 820 (36.49)	42 107 (37.57)	13 572 (38.75)	8206 (40.67)	
White collar, *N* (*%*)	263 805 (41.33)	197 830 (42.01)	45 154 (40.28)	13 465 (38.45)	7356 (36.46)	
Own business, *N* (*%*)	104 999 (16.45)	76 390 (16.22)	18 928 (16.89)	6146 (17.55)	3555 (17.62)	
Unknown, *N* (*%*)	33 704 (5.28)	24 903 (5.29)	5900 (5.26)	1840 (5.25)	1061 (5.26)	<0.001
Household Crowding						
Overcrowded, *N* (*%*)	180 539 (28.29)	130 274 (27.66)	33 368 (29.77)	10 582 (30.21)	6315 (31.30)	
Not overcrowded, *N* (*%*)	448 877 (70.33)	334 097 (70.95)	77 188 (68.86)	23 973 (68.45)	13 619 (67.49)	
Unknown, *N* (*%*)	8797 (1.38)	6552 (1.39)	1533 (1.37)	468 (1.34)	244 (1.21)	<0.001

a *p* values are based on ANOVA tests for continuous and χ^2^ tests for categorical variables.

### Associations between IQ, schizophrenia, and ONAP

With each one-point increase in IQ, risk decreased for schizophrenia (unadjusted HR = 0.963, 95% CI 0.961–0.964; adjusted HR = 0.961, 95% CI 0.960–0.963), and for ONAP (unadjusted HR = 0.974; 95% CI 0.972–0.976; adjusted HR = 0.973, 95% CI 0.971–0.975). Evidence for these associations remained after excluding individuals hospitalised within 2 years after conscription (online Supplementary Table 1).

### Association between ESR and IQ

Higher ESR was associated with lower IQ at the time of conscription in a dose-response function (Table 2). When compared with ESR of 0–3 mm/h, there was a small but robust decrease in IQ for groups with higher ESR levels. After adjusting for potential confounders these associations attenuated, but remained significant. We explored whether the dose–response relationship observed using ESR as a categorical variable could be more parsimoniously explained using ESR as a continuous variable. Comparing these models using a log-likelihood test revealed no significant difference between two models (*p* = 0.62) suggesting that categorical classification of ESR is not superior to a linear description of the ESR-IQ association (online Supplementary Table 2).

**Table 2.** Association between ESR and IQ at age 18 years

ESR (mm/h) at 18–20 years	No.	Mean IQ at 18–20 years (SD)	Mean difference [95% CI]^a^			
			Unadjusted	*p* Value	Adjusted^b^	*p* Value
0–3	470 923	100.20 (15.00)	Reference	−	Reference	−
4–6	112 089	99.71 (14.96)	−0.48 [−0.58, −0.38]	<0.001	−0.30 [−0.39, −0.20]	<0.001
7–10	35 023	99.23 (15.04)	−0.95 [−1.12, −0.79]	<0.001	−0.64 [−0.80, −0.49]	<0.001
⩾11	20 178	98.61 (14.98)	−1.57 [−1.78, −1.36]	<0.001	−1.08 [−1.28, −0.88]	<0.001

a The mean difference was calculated using the group with ESR = 0–3 mm/h as a reference.

b Regression models were adjusted for household crowding, winter birth, parental socioeconomic status at 8–12 years, migration status, and parental history of non-affective psychoses.

### Associations between ESR, schizophrenia, and ONAP

**Fig. 1.**
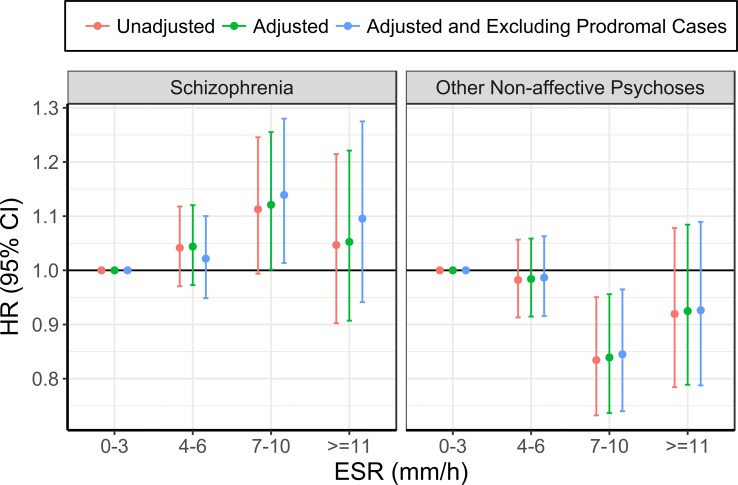
Association between ESR and schizophrenia (left), and other non-affective psychoses (right) (the group with ESR of 0–3 mm/h was used as the reference for all analyses).

The risk of future hospitalisation with schizophrenia was higher in the group with ESR of 7–10 mm/h at baseline, compared with 0–3 mm/h, although this association only reached statistical significance after adjusting for confounding variables (see Table 3; Figure 1; online Supplementary Table 3). Conversely, the risk for ONAP was significantly lower in the group with ESR of 7–10 mm/h, compared with 0–3 mm/h, both before and after adjusting for confounders. There were no significant differences between ESR bands of 4–6 mm/h and ⩾11 mm/h as compared with the reference group (0–3 mm/h). Exclusion of prodromal cases did not change the pattern of results.

**Table 3.** Association between ESR and subsequent schizophrenia and other non-affective psychoses

ESR (mm/h)	Unadjusted analyses				After adjustment for potential confounders^a^ and exclusion of cases diagnosed within 2 years of conscription			
	No. (% of total)^b^	Case, No. (%)	HR [95% CI]	*p* value	No. (% of total)^b^	Case, No. (%)	HR [95% CI]	*p* value
Schizophrenia								
0–3	467 073 (73.78)	3932 (0.84)	1.00 [reference]	−	466 774 (73.78)	3633 (0.78)	1.00 [reference]	−
4–6	111 204 (17.57)	958 (0.86)	1.04 [0.97, 1.12]	0.258	111 112 (17.56)	866 (0.78)	1.02 [0.95, 1.10]	0.573
7–10	34 783 (5.49)	326 (0.94)	1.11 [0.99, 1.25]	0.064	34 763 (5.49)	306 (0.88)	1.14 [1.01, 1.28]	0.029*
⩾11	20 020 (3.16)	182 (0.91)	1.05 [0.90, 1.22]	0.546	20 013 (3.16)	175 (0.87)	1.10 [0.94, 1.28]	0.239
Other non-affective psychoses								
0–3	466 991 (73.80)	3850 (0.82)	1.00 [reference]	−	466 835 (73.79)	3694 (0.79)	1.00 [reference]	−
4–6	111 131 (17.56)	885 (0.80)	0.98 [0.91, 1.06]	0.629	111 079 (17.56)	851 (0.77)	0.99 [0.92, 1.06]	0.722
7–10	34 697 (5.48)	240 (0.69)	0.83 [0.73, 0.95]	0.006*	34 689 (5.48)	232 (0.67)	0.85 [0.74, 0.96]	0.013*
⩾11	19 996 (3.16)	158 (0.79)	0.92 [0.78, 1.08]	0.301	19 990 (3.16)	152 (0.76)	0.93 [0.79, 1.09]	0.355

a Cox regression models were adjusted for household crowding, winter birth, parental socioeconomic status at 8–12 years, migration status, and parental history of non-affective psychoses.

b Cases of other non-affective psychosis were excluded from the analysis of schizophrenia and *vice versa*.

* *p* < 0.05

### Co-relative control analysis of the ESR-psychoses association

The HRs for schizophrenia and ONAP for the group with ESR levels 7–10 mm/h, compared with 0–3 mm/h, was similar for the general population and full-sibling pairs (who share on average 50% of their genomes and presumably the largest number of environmental factors among the investigated groups of co-relatives): higher ESR *increased* risk of schizophrenia, but *decreased* risk of ONAP (Table 4). The results offer some support that these specific ESR and psychoses relationships may not be fully confounded by shared familial factors.

**Table 4.** Co-relative analyses for association between ESR, schizophrenia and other non-affective psychoses

	HR [95% CI]* for schizophrenia and other non-affective psychoses							
	Schizophrenia				Other non-affective psychoses			
	No.	ESR 4–6 mm/h	ESR 7–10 mm/h	ESR ⩾11 mm/h	No.	ESR 4–6 mm/h	ESR 7–10 mm/h	ESR ⩾11 mm/h
General population	633 080	1.04 [0.97, 1.12]	1.11 [0.99, 1.25]	1.05 [0.90, 1.22]	632 815	0.98 [0.91, 1.06]	0.83 [0.73, 0.95]	0.92 [0.78, 1.08]
Cousins	61 091	0.78 [0.61, 0.99]	0.87 [0.57, 1.34]	1.14 [0.67, 1.95]	61 153	0.97 [0.77, 1.22]	0.71 [0.47, 1.08]	0.80 [0.49, 1.31]
Half-siblings	21 264	1.08 [0.76, 1.52]	1.50 [0.86, 2.61]	1.11 [0.53, 2.34]	21 239	1.05 [0.73, 1.52]	1.02 [0.58, 1.80]	1.01 [0.38, 2.70]
Full-siblings	109 166	0.91 [0.76, 1.08]	1.39 [1.04, 1.85]	0.84 [0.59, 1.21]	109 115	0.82 [0.68, 0.99]	0.70 [0.51, 0.95]	1.14 [0.76, 1.71]

* The group with ESR 0–3 mm/h has been used as the reference category for all analyses

### Co-relative control analysis of the ESR-IQ association

Co-relative control analysis of the ESR-IQ association, using linear mixed-effects regression and a random intercept for relative pairs, showed evidence of familial confounding (online Supplementary Table 4). The mean difference in IQ for higher ESR levels (compared with ESR 0–3 mm/h) were between 0.5 and 1.5 IQ points in the general population (ESR 4–6 mm/h = −0.48, 95% CI −0.58 to −0.38; ESR 7–10 mm/h = −0.95, 95% CI −1.12 to −0.79; ESR ⩾11 mm/h = −1.57, 95% CI −1.78 to −1.36). However, this difference markedly decreased in a gradual manner with increasing family relatedness (results for full-sibling pairs: ESR 4–6 mm/h = −0.02, 95% CI −0.17 to 0.14; ESR 7–10 mm/h = −0.21, 95% CI −0.44 to 0.03; ESR ⩾11 mm/h = −0.68, 95% CI −0.98 to −0.38.

### Mediation and moderation of the ESR and psychoses association by IQ

We tested whether IQ mediated the association of ESR (7–10 mm/h) with schizophrenia and ONAP. For schizophrenia, the indirect effect (mediated by IQ) was significant in the adjusted model (*β* = 0.004, se < 0.001, *p* < 0.001). Similarly, for ONAP, the indirect effect was significant in both unadjusted (*β* = 0.003, se < 0.001, *p* < 0.001) and adjusted models (*β* = 0.003, se < 0.001, *p* < 0.001; see online Supplementary Table 5 and online Supplementary Fig. 2). However, there was no evidence for an interaction between ESR and IQ on regression models for schizophrenia or ONAP (online Supplementary Table 6).

### Sensitivity analyses of ESR and psychoses association

We re-examined the ESR-psychosis association using ESR as a continuous variable, using ESR quartiles, and using ESR deciles before and after excluding participants with a suspected acute infection (ESR >22 mm/h). After adjusting for potential confounders and excluding both potentially prodromal cases of psychosis and those with suspected infection, there was trend level association indicating that each one-point increase in ESR increased the risk of schizophrenia (*p* = 0.070), but decreased the risk of ONAP (*p* = 0.053); see online Supplementary Table 7.

Using ESR as quartiles, there was no association with schizophrenia (online Supplementary Table 8). However, those in the 3rd quartile of ESR had an increased risk of ONAP compared with 1st quartile; unadjusted HR 1.09 (95% CI, 1.01, 1.17); *p* = 0.029; evidence for this association attenuated after adjusting for potential confounders and exclusions (*p* = 0.074) (online Supplementary Table 9).

After adjusting for potential confounders and exclusions, there was a trend level association indicating that those in the top decile of ESR, compared with bottom decile, had an increased risk of schizophrenia (HR = 1.12; 95% CI, 1.00, 1.27); *p* = 0.056 (online Supplementary Table 10), but decreased risk of ONAP (HR = 0.89; 95% CI, 0.78, 1.01); *p* = 0.074 (online Supplementary Table 11).

## Discussion

The present study investigated potential roles of inflammation and neurodevelopment in the pathogenesis of psychotic disorders in a large Swedish population-based male sample. We showed that lower IQ is robustly associated with subsequent diagnoses of schizophrenia and ONAP. We also observed a cross-sectional association between ESR and IQ that followed a linear pattern; co-relative control analyses showed this association was partly due to familial factors. There was some, albeit weak, evidence for an association between ESR and increased risk for schizophrenia. Contrary to our hypotheses, higher ESR was associated with decreased risk for ONAP. Co-relative control analyses suggested that respective associations between ESR and subsequent risk of psychosis might not be explained fully by shared familial factors. With regard to the interplay between inflammation and neurodevelopment, there was some evidence that IQ partly mediated the association between ESR and psychotic disorders. There was no evidence for interaction between ESR and IQ with regard to the risk of psychoses.

The association between premorbid IQ and psychotic disorders has been studied extensively. Our findings are consistent with previous studies reporting lower premorbid IQ in future patients of schizophrenia and ONAP (Jones *et al.* 1994; Crow *et al.* 1995; David *et al.* 1997; Davidson *et al.* 1999; Cannon *et al.* 2000, 2002; Zammit *et al.* 2004; Reichenberg *et al.* 2005; Kendler *et al.* 2015). Adjusting for potential confounders and exclusion of suspected prodromal cases had minimal impact on this association. Previous studies indicate unique environmental factors may contribute to lower premorbid IQ in psychotic disorders (Kendler *et al.* 2015, 2016*a*). Our work indicates that low-grade inflammation may be one such factor.

We have presented separate analyses for schizophrenia and ONAP to cover the entire spectrum of psychotic disorders. In young people, diagnosis can change over time, e.g. from other NAP to schizophrenia (Fusar-Poli *et al.* 2016). Longitudinal studies have reported that lower premorbid IQ (Kendler *et al.* 2016*b*), prenatal/ childhood infections (Brown *et al.* 2004; Dalman *et al.* 2008; Blomstrom *et al.* 2016) are associated with schizophrenia and ONAP suggesting impaired neurodevelopment and immune-related risk factors are relevant for all of these disorders.

The results for an association between ESR and IQ are consistent with our previous Swedish population-based study based on a smaller sample of 49 321 participants (Karlsson *et al.* 2010). The findings indicate that low-grade systemic inflammation, as indexed by ESR, might affect cognitive development/functioning. However, the cross-sectional nature of the ESR-IQ association means that reverse causality cannot be excluded (Luciano *et al.* 2009). In future, longitudinal studies are required. Cognitive impairments associated with acute inflammatory conditions observed in experimental studies of humans and animals may be part of a ‘sickness response’, which is mediated by effector molecules such as cytokines (Reichenberg *et al.* 2001). Chronic low-grade inflammation, as indexed by elevated concentrations of inflammatory markers, has also been associated with cognitive impairments in elderly individuals (Gorelick, 2010) and with lower IQ in children (MacKinnon *et al.* 2017). In the elderly, such effects may involve vascular changes in the brain parenchyma induced by subtle but chronic elevations in inflammatory mediators associated with aging and other factors (Casserly & Topol, 2004). In children, however, potential mechanisms are less clear, and might involve effects of inflammatory cytokines on brain development and functioning (Spencer *et al.* 2005; Asp *et al.* 2009; McAfoose & Baune, 2009; Liu *et al.* 2014; Khandaker & Dantzer, 2015).

Low-grade inflammation had an opposing effect on different types of psychotic disorders; higher ESR increased the risk of schizophrenia but decreased the risk of ONAP. A Previous study has shown that higher levels of IL-6 at age 9 are associated with increased risk for psychotic symptoms at 18 years (Khandaker *et al.* 2014). Other studies have reported an association between higher levels of CRP at baseline and subsequent risk for schizophrenia at follow-up (Wium-Andersen *et al.* 2013; Metcalf *et al.* 2017). These findings are consistent with our results that ESR levels in the upper normal range are associated with increased risk for schizophrenia. Protective effect of high ESR for ONAP in our sample might be due to diverse diagnoses included in this group. In a Finnish longitudinal cohort study higher CRP in adolescence was associated with increased risk of schizophrenia, but not ONAP, in adulthood (Metcalf *et al.* 2017). We previously reported an association between lower concentrations of acute phase proteins in neonatal blood spots and diagnosis of non-affective psychosis including schizophrenia in adulthood; but did not have power to examine schizophrenia and other psychosis separately (Gardner *et al.* 2013). Taken together, the current studies indicate that low-grade inflammation may increase the risk of schizophrenia, but its association with ONAP is less clear, indicating that further studies looking at specific types of psychoses are required.

Results from co-relative control analyses further refine our results of the ESR-psychoses and ESR-IQ associations. The association between ESR and schizophrenia was comparable between individuals in the general population and between members of full-sibling pairs, suggesting that shared familial factors are unlikely to explain these associations fully. This suggests unique environmental exposures rather than genetic or shared environmental factors may be important. This is consistent with a Danish population-based study that reported that polygenic risk scores for schizophrenia and a history of infections were independent risk-factors for schizophrenia (Benros *et al.* 2016). As the results for cousin and half-sibling pairs were not significant, this analysis requires replication in a different, possibly even larger sample. The ESR-IQ association, however, clearly highlighted familial influence on this association. Population-based studies do suggest that susceptibility to infection may have a genetic contribution (Cooke & Hill, 2001; Obel *et al.* 2010; Hwang *et al.* 2012), so the association of systemic inflammation and cognitive functioning might be largely based on genetic, but also shared environmental factors. Thus, our findings provide an interesting distinction: the association of low-grade inflammation with cognitive functioning might be due to shared familial factors, but its association with psychosis may be due to unique, environmental factors.

The results of our mediation analysis indicate that inflammation may influence the risk of psychosis by affecting neurodevelopment. Thus, immune and neurodevelopmental mechanisms for pathogenesis of psychosis could be potentially intertwined. ESR increased the risk for both schizophrenia and ONAP (indirectly via IQ), yet also lowered risk (directly) for ONAP. Statistically, this phenomenon has been described as suppression (MacKinnon *et al.* 2000), in which both risk and protective factors operate together. Suppression hints towards the presence of different, opposing biological pathways, where only the sum of the direct and indirect effects of a risk factor reflects its final contribution towards causation of an illness.

The results of this study need to be considered in light of its limitations. ESR is a marker of systemic inflammation and it represents activation of acute phase response due to various stimuli (e.g., infection, cancer). In future, studies of more sensitive and direct measures of innate immune activity, such as IL-6 or CRP, might be helpful. We carried out a number of sensitivity analyses to explore the ESR-psychosis relationships further. There was some evidence for an association between ESR and schizophrenia for participants in the top decile of ESR distribution but effects were not robust. The analyses presented here are based on male conscripts and might not be generalisable to women. However, previous studies did not indicate any notable sex difference for the association between inflammation and cognition (Khandaker *et al.* 2015*b*; Gale *et al.* 2016), or for the association between inflammation and psychoses (Khandaker *et al.* 2014). Strengths of this study include a large population-based sample with data from reliable general population registers, long follow-up period, and innovative co-relative analyses that allowed exploration of shared familial confounding.

In conclusion, we report that lower IQ is associated with low-grade inflammation (cross-sectional), and with an increased risk of psychotic disorders (longitudinal) in adulthood. There is some evidence that inflammation may increase the risk of schizophrenia by affecting processes involved in cognitive development.

## Supplementary material

For supplementary material accompanying this paper visit https://doi.org/10.1017/S0033291718000831. click here to view supplementary material
