# Supplementary material for: Systemic inflammation and intelligence in early adulthood and subsequent risk of schizophrenia and other non-affective psychoses: a longitudinal cohort and co-relative study
Source: Psychol Med. 2018 Apr 6;49(2):295–302. doi: 10.1017/S0033291718000831 (PMC6316362; doi:10.1017/S0033291718000831)
Supplement: Supplementary file 1 [file S0033291718000831sup001.docx]

**Kappelmann *et. al.* Online Supplementary Material**

**Materials and Methods**

**Study population**

Online Supplementary Figure 1:


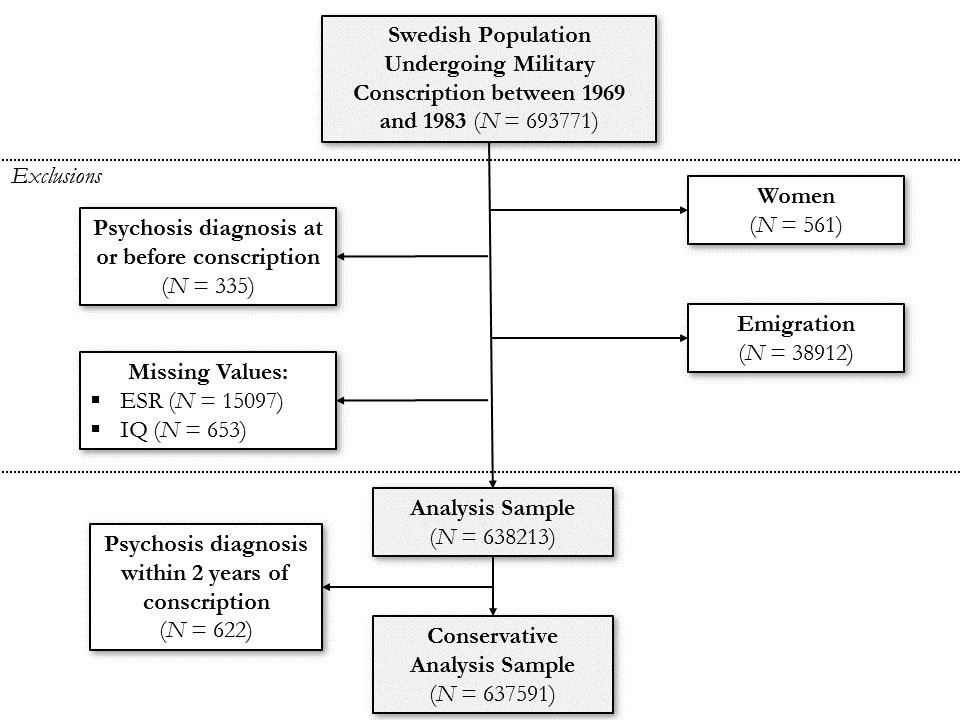


*Figure Caption:* Participation of women in military service was voluntary and not compulsory.

**Results**

**Relationship of IQ and Psychoses**

Online Supplementary Table 1: Analysis of Association between IQ and the Development of Psychoses

|  | Schizophrenia | | | | Other Non-Affective psychoses | | | |
| --- | --- | --- | --- | --- | --- | --- | --- | --- |
|  | Total Sample, No. | No. of cases (%) | HR (IQ+1) [95% CI] | P-value | Total Sample, No. | No. of cases (%) | HR (IQ+1) [95% CI] | P-value |
| Unadjusted estimates | 633080 | 5398 (0.85) | 0.963 [0.961, 0.964] | <0.001 | 632815 | 5133 (0.81) | 0.974 [0.972, 0.976] | <0.001 |
| Adjusted estimates† | 633080 | 5398 (0.85) | 0.961 [0.960, 0.963] | <0.001 | 632815 | 5133 (0.81) | 0.973 [0.971, 0.975] | <0.001 |
| Adjusted estimates excluding patients diagnosed within 2 years† | 632662 | 4980 (0.79) | 0.962 [0.960, 0.964] | <0.001 | 632611 | 4929 (0.78) | 0.973 [0.971, 0.975] | <0.001 |

^1^ Adjustments have been made for household crowding, winter birth, parental socioeconomic status at 8-12 years, migration status, and parental history of non-affective psychoses.

**Association of ESR and IQ**

Online Supplementary Table 2: Test of Linearity for Association between IQ and ESR

| Model | Residual Degrees of Freedom | Residual Sum of Squares | Degrees of Freedom | Sum of Squares | P-Value |
| --- | --- | --- | --- | --- | --- |
| Dummy model^1^ | 638211 | 143512579 |  |  |  |
| Linear model^2^ | 638209 | 143512361 | 2 | 218.56 | 0.615 |

^1^ The dummy model codes ESR in bands 0-3, 4-6, 7-10, and ≥11mm/h as done in other analyses.

^2^ The linear model re-codes ESR bands of 0-3, 4-6, 7-10, and ≥11mm/h into values -1.5, -0.5, 0.5, and 1.5, respectively.

**Relationship of ESR and Psychoses**

Online Supplementary Table 3: Analysis of the Association between ESR and Subsequent Non-Affective Psychoses

| ESR (mm/h) | No. (% of total)^1^ | Case, No. (%) | Unadjusted | | Adjusted^2^ | |
| --- | --- | --- | --- | --- | --- | --- |
|  |  |  | HR [95% CI] | P-value | HR [95% CI] | P-value |
| Schizophrenia | | | | | | |
| 0-3 | 467073 (73.78) | 3932 (0.84) | 1.00 [reference] | - | 1.00 [reference] | - |
| 4-6 | 111204 (17.57) | 958 (0.86) | 1.04 [0.97, 1.12] | 0.258 | 1.04 [0.97, 1.12] | 0.234 |
| 7-10 | 34783 (5.49) | 326 (0.94) | 1.11 [0.99, 1.25] | 0.064 | 1.12 [1.00, 1.26] | 0.048^*^ |
| ≥11 | 20020 (3.16) | 182 (0.91) | 1.05 [0.90, 1.22] | 0.546 | 1.05 [0.91, 1.22] | 0.500 |
| Other Non-Affective Psychoses | | | | | | |
| 0-3 | 466991 (73.80) | 3850 (0.82) | 1.00 [reference] | - | 1.00 [reference] | - |
| 4-6 | 111131 (17.56) | 885 (0.80) | 0.98 [0.91, 1.06] | 0.629 | 0.98 [0.91, 1.06] | 0.667 |
| 7-10 | 34697 (5.48) | 240 (0.69) | 0.83 [0.73, 0.95] | 0.006^*^ | 0.84 [0.74, 0.96] | 0.008^*^ |
| ≥11 | 19996 (3.16) | 158 (0.79) | 0.92 [0.78, 1.08] | 0.301 | 0.92 [0.79, 1.08] | 0.335 |

^1^ Cases of other non-affective psychosis were excluded from the analysis of schizophrenia and *vice versa*.

^2^ Adjustments have been made for household crowding, winter birth, parental socioeconomic status at 8-12 years, migration status, and parental history of non-affective psychoses.

^*^ P<0.05.

**Co-relative Control Analysis of the ESR-IQ Association**

Online Supplementary Table 4: Co-Relative Analyses using Linear Mixed-Effects Regression for Association between ESR and IQ

|  | Mean Difference in IQ [95% CI]* | | | |
| --- | --- | --- | --- | --- |
|  | No. | 4-6mm/h | 7-10mm/h | ≥11mm/h |
| General population | 638213 | -0.48 [-0.58, -0.38] | -0.95 [-1.12, -0.79] | -1.57 [-1.78, -1.36] |
| Cousins | 61590 | -0.09 [-0.33, 0.14] | -0.70 [-1.05, -1.56] | -1.11 [-1.56, -0.66] |
| Half siblings | 21488 | 0.29 [-0.09, 0.68] | -0.04 [-0.61, 0.53] | -0.96 [-1.68, -0.24] |
| Full siblings | 110010 | -0.02 [-0.17, 0.14] | -0.21 [-0.44, 0.03] | -0.68 [-0.98, -0.38] |

* The group with ESR 0-3mm/h has been used as reference category for all analyses.

**Mediation/Moderation of the ESR and Psychoses Relationship by IQ**

Online Supplementary Table 5: Mediation of the ESR and Psychoses Relationship by IQ

|  | Schizophrenia^2^ | | Other Non-Affective Psychoses^2^ | |
| --- | --- | --- | --- | --- |
|  | Standardised effect (standard error) | P-Value | Standardised effect (standard error) | P-Value |
| Unadjusted |  |  |  |  |
| Direct effect | Criteria not met^1^ | - | -0.028 (0.009) | 0.002 |
| Indirect effect via IQ at 18-20 years | Criteria not met^1^ | ^-^ | 0.003 (<0.001) | <0.001 |
| Total effect | Criteria not met^1^ | - | -0.024 (0.009) | 0.007 |
| Adjusted^3^ |  |  |  |  |
| Direct effect | 0.013 (0.007) | 0.070 | -0.026 (0.010) | 0.008 |
| Indirect effect via IQ at 18-20 years | 0.004 (<0.001) | <0.001 | 0.003 (<0.001) | <0.001 |
| Total effect | 0.018 (0.007) | 0.015 | -0.023 (0.010) | 0.008 |

^1^ Mediation of the ESR and schizophrenia relationship was not calculated as the unadjusted main effect of ESR on schizophrenia did not reach significance, which is a prerequisite for mediation.

^2^ Since effects of ESR on psychoses were specific to the difference between ESR of 0-3mm/h and ESR of 7-10mm/h in main analyses, the sample for mediation analysis was reduced to individuals falling into these ESR bands only. Furthermore, individuals with respective other diagnosis were excluded as well as individuals diagnosed within 2 years for adjusted analyses. Final sample sizes were: n=505946 for adjusted schizophrenia analysis and n=505946 for unadjusted and 501524 for adjusted analyses of other non-affective psychoses.

^3^ Adjustments have been made for household crowding, winter birth, parental socioeconomic status at 8-12 years, migration status, and parental history of non-affective psychoses and by excluding individuals diagnosed within 2 years of conscription.

Online Supplementary Figure 2:


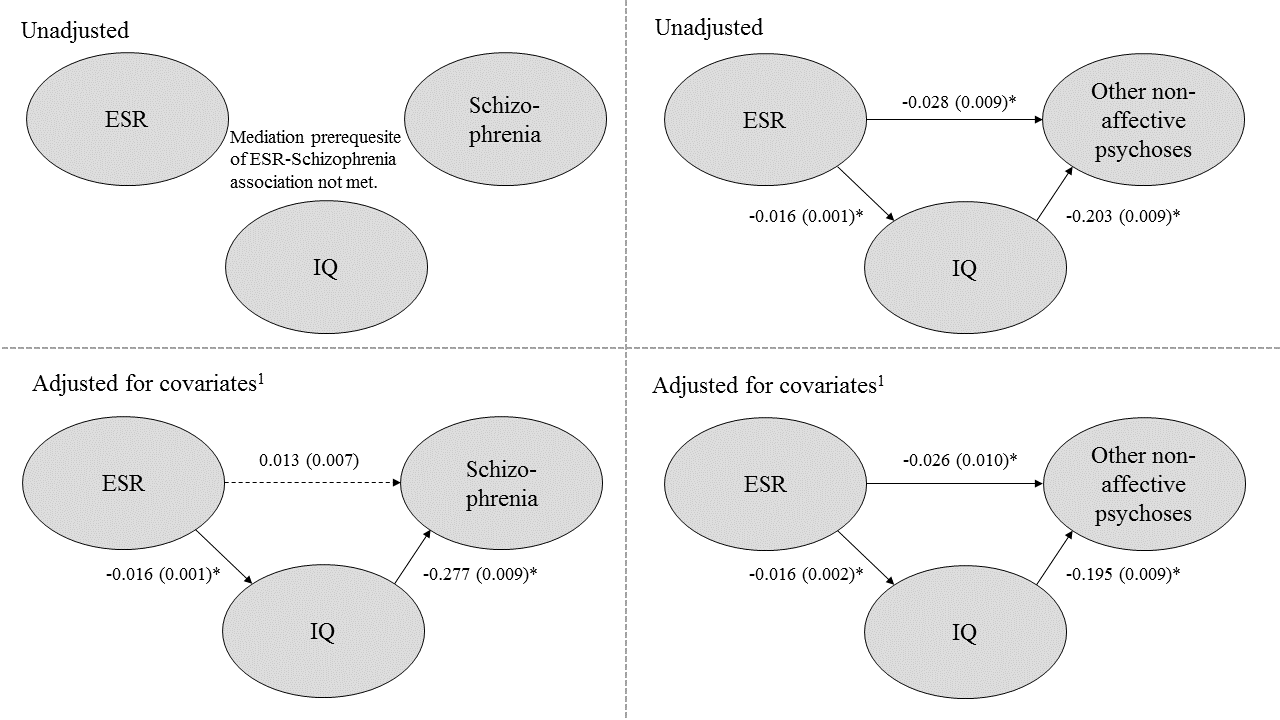


^1^ Adjustments have been made for household crowding, winter birth, parental socioeconomic status at 8-12 years, migration status, and parental history of non-affective psychoses and by excluding individuals diagnosed within 2 years of conscription.

^*^ Significance at P<0.05 is indicated by * and by non-dashed arrows.

Online Supplementary Table 6: Interaction Analyses of ESR and IQ on Non-Affective Psychoses

|  | Schizophrenia | | | Other Non-Affective Psychoses | | |
| --- | --- | --- | --- | --- | --- | --- |
| N=638213 | Β (SE) | OR [95% CI] | P-value | Β (SE) | OR [95% CI] | P-value |
| Intercept | -4.908 (0.018) | - | <0.001^*^ | -4.852 (0.017) | - | <0.001^*^ |
| ESR: 4-6mm/h | -0.010 (0.042) | 0.990 [0.911, 1.074] | 0.816 | -0.044 (0.040) | 0.957 [0.884, 1.034] | 0.271 |
| ESR: 7-10mm/h | 0.015 (0.070) | 1.015 [0.881, 1.161] | 0.832 | -0.256 (0.076) | 0.774 [0.665, 0.895] | <0.001^*^ |
| ESR: ≥11mm/h | 0.038 (0.090) | 1.039 [0.867, 1.231] | 0.668 | -0.120 (0.092) | 0.887 [0.736, 1.058] | 0.196 |
| IQ^1^ | 0.037 (0.001) | 1.037 [1.035, 1.040] | <0.001^*^ | 0.025 (0.001) | 1.025 [1.023, 1.028] | <0.001^*^ |
| ESR: 4-6mm/h * IQ^2^ | 0.002 (0.003) | 1.002 [0.997, 1.007] | 0.420 | -0.000 (0.003) | 1.000 [0.995, 1.005] | 0.874 |
| ESR: 7-10mm/h * IQ^2^ | 0.006 (-0.002) | 1.006 [0.998, 1.014] | 0.118 | 0.008 (0.005) | 1.008 [0.999, 1.017] | 0.086 |
| ESR: ≥11mm/h * IQ^2^ | -0.002 (0.005) | 0.998 [0.988, 1.009] | 0.735 | 0.005 (0.006) | 1.005 [0.994, 1.016] | 0.352 |

^1^ IQ has been centered, to *M*=0 and *SD*=15, and reversed for these analyses to allow interpretation of the ESR main effects as well as the interaction.

^2^ The interaction ORs specify the added OR of non-affective psychoses for every 1-point decrease in IQ in individuals exposed to the respective higher ESR level on top of the main effect ORs.

^*^ P<0.05.

**Sensitivity Analyses of the ESR-Psychoses Association**

Online Supplementary Table 7: Sensitivity Analyses – Hazard Ratio (HR) for Psychotic Disorders for Each One-Point Increase in ESR

| Regression Models | Schizophrenia | | | | Other Non-affective Psychoses | | | |
| --- | --- | --- | --- | --- | --- | --- | --- | --- |
|  | No. | Case, No. | HR (95% CI) | P-Value | No. | Case, No. | HR (95% CI) | P-Value |
| Unadjusted analysis | 633080 | 5398 | 1.002 [0.994, 1.010] | 0.661 | 632815 | 5133 | 0.995 [0.987, 1.004] | 0.269 |
| After adjusting for potential confounders and excluding participants diagnosed with psychosis within 2 years of conscription^1^ | 632662 | 4980 | 1.004 [0.996, 1.012] | 0.337 | 632611 | 4929 | 0.996 [0.988, 1.005] | 0.363 |
| After adjusting for potential confounders, excluding participants diagnosed with psychosis within 2 years of conscription, and excluding those with suspected infection (ESR>22mm/h)^1^ | 629691 | 4958 | 1.009 [0.999, 1.019] | 0.070 | 629633 | 4900 | 0.990 [0.979, 1.000] | 0.053 |

^1^ Regression models were adjusted for household crowding, winter birth, parental socioeconomic status at 8-12 years, migration status, and parental history of non-affective psychoses.

Online Supplementary Table 8: Sensitivity Analyses – Hazard Ratios (HR) for Schizophrenia for Quartiles of ESR

| ESR | | Unadjusted estimates (n=633080) | | After adjusting for potential confounders and excluding participants diagnosed with psychosis within 2 years of conscription (n=632662)^1^ | | After adjusting for potential confounders, excluding participants diagnosed with psychosis within 2 years of conscription, and excluding those with suspected infection (ESR>22mm/h; n=629691)^1^ | |
| --- | --- | --- | --- | --- | --- | --- | --- |
| Quartile | Range (mm/h) | HR [95% CI] | P-Value | HR (95% CI) | P-Value | HR (95% CI) | P-Value |
| 1 | 0.00-1.87 | 1 [reference] | - | 1 [reference] | - | 1 [reference] | - |
| 2 | 1.87-2.18 | 1.00 [0.92, 1.08] | 0.944 | 1.01 [0.93, 1.09] | 0.887 | 1.01 [0.93, 1.09] | 0.883 |
| 3 | 2.18-4.00 | 1.03 [0.95, 1.11] | 0.480 | 1.04 [0.96, 1.12] | 0.384 | 1.04 [0.96, 1.12] | 0.381 |
| 4 | 4.00-95.8^2^ | 1.05 [0.97, 1.13] | 0.211 | 1.05 [0.97, 1.14] | 0.191 | 1.06 [0.98, 1.14] | 0.177 |

^1^ Regression models were adjusted for household crowding, winter birth, parental socioeconomic status at 8-12 years, migration status, and parental history of non-affective psychoses.

^2^ After the exclusion of individuals with ESR>22mm/h, the total range of ESR values for this quartile reduced to 6.40-19.99mm/h.

Online Supplementary Table 9: Sensitivity Analyses – Hazard Ratios (HR) for Other Non-affective Psychoses Association for ESR Quartiles

| ESR | | Unadjusted analysis (n=632815) | | After adjusting for potential confounders and excluding participants diagnosed with psychosis within 2 years of conscription (n=632611)^1^ | | After adjusting for potential confounders, excluding participants diagnosed with psychosis within 2 years of conscription, and excluding those with suspected infection (ESR>22mm/h; n=629633)^1^ | |
| --- | --- | --- | --- | --- | --- | --- | --- |
| Quartile | Range (mm/h) | HR [95% CI] | P-Value | HR (95% CI) | P-Value | HR (95% CI) | P-Value |
| 1 | 0.00-1.87 | 1 [reference] | - | 1 [reference] | - | 1 [reference] | - |
| 2 | 1.87-2.18 | 0.99 [0.91, 1.06] | 0.707 | 0.98 [0.90, 1.06]^3^ | 0.533^3^ | 0.98 [0.90, 1.06] ^3^ | 0.533^3^ |
| 3 | 2.18-4.00 | 1.09 [1.01, 1.17] | 0.029 | 1.07 [0.99, 1.16] | 0.074 | 1.07 [0.99, 1.16] | 0.074 |
| 4 | 4.00-95.8^2^ | 0.94 [0.87, 1.02] | 0.145 | 0.94 [0.87, 1.02] | 0.161 | 0.94 [0.87, 1.02] | 0.125 |

^1^ Regression models were adjusted for household crowding, winter birth, parental socioeconomic status at 8-12 years, migration status, and parental history of non-affective psychoses.

^2^ After the exclusion of individuals with ESR>22mm/h, the total range of ESR values for this quartile reduced to 6.40-22mm/h.

^3^ The proportional hazards assumption for this predictor was violated.

Online Supplementary Table 10: Sensitivity Analyses – Hazard Ratios (HR) for Schizophrenia for ESR Deciles

| ESR | | Unadjusted analysis (n=633080) | | After adjusting for potential confounders and excluding participants diagnosed with psychosis within 2 years of conscription (n=632662)^1^ | | After adjusting for potential confounders, excluding participants diagnosed with psychosis within 2 years of conscription, and excluding those with suspected infection (ESR>22mm/h; n=629691)^1^ | |
| --- | --- | --- | --- | --- | --- | --- | --- |
| Decile | Range (mm/h) | HR [95% CI] | P-Value | HR [95% CI] | P-Value | HR (95% CI) | P-Value |
| 1 | 0.00-1.04 | 1 [reference] | - | 1 [reference] | - | 1 [reference] | - |
| 2 | 1.04-1.11 | 0.97 [0.87, 1.09] | 0.657 | 0.95 [0.85, 1.07] | 0.404 | 0.95 [0.85, 1.07] | 0.403 |
| 3 | 1.11-2.00 | 0.98 [0.87, 1.10] | 0.706 | 0.98 [0.87, 1.10] | 0.720 | 0.98 [0.87, 1.10] | 0.722 |
| 4 | 2.00-2.09 | 0.98 [0.87, 1.11] | 0.756 | 0.96 [0.85, 1.09] | 0.529 | 0.96 [0.85, 1.09] | 0.531 |
| 5 | 2.09-2.18 | 1.05 [0.92, 1.19] | 0.480 | 1.04 [0.91, 1.18] | 0.594 | 1.04 [0.91, 1.18] | 0.593 |
| 6 | 2.18-3.00 | 1.03 [0.92, 1.16] | 0.597 | 1.03 [0.91, 1.16] | 0.632 | 1.03 [0.91, 1.16] | 0.631 |
| 7 | 3.00-3.33 | 0.99 [0.88, 1.12] | 0.865 | 0.98 [0.86, 1.11] | 0.716 | 0.98 [0.86, 1.11] | 0.718 |
| 8 | 3.33-4.44 | 1.06 [0.95, 1.20] | 0.303 | 1.05 [0.93, 1.18] | 0.470 | 1.05 [0.93, 1.18] | 0.468 |
| 9 | 4.44-6.40 | 0.97 [0.86, 1.10] | 0.662 | 0.94 [0.83, 1.06] | 0.328 | 0.94 [0.83, 1.07] | 0.330 |
| 10 | 6.40-95.80^2^ | 1.10 [0.98, 1.23] | 0.114 | 1.12 [0.99, 1.26] | 0.072 | 1.12 [1.00, 1.27] | 0.056 |

^1^ Regression models were adjusted for household crowding, winter birth, parental socioeconomic status at 8-12 years, migration status, and parental history of non-affective psychoses.

^2^ After the exclusion of individuals with ESR>22mm/h, the total range of ESR values for this decile reduced to 6.40-19.99mm/h.

Online Supplementary Table 11: Sensitivity Analyses – Hazard Ratios (HR) for Other Non-affective Psychoses for ESR Deciles

| ESR | | Unadjusted analysis (n=632815) | | After adjusting for potential confounders and excluding participants diagnosed with psychosis within 2 years of conscription(n=632611)^1^ | | After adjusting for potential confounders, excluding participants diagnosed with psychosis within 2 years of conscription, and excluding those with suspected infection (ESR>22mm/h; n=629633)^1^ | |
| --- | --- | --- | --- | --- | --- | --- | --- |
| Decile | Range (mm/h) | HR [95% CI] | P-Value | HR [95% CI] | P-Value | HR (95% CI) | P-Value |
| 1 | 0.00-1.04 | 1 [reference] | - | 1 [reference] | - | 1 [reference] | - |
| 2 | 1.04-1.11 | 0.97 [0.86, 1.09] | 0.580 | 0.98 [0.87, 1.10] | 0.713 | 0.98 [0.87, 1.10] | 0.713 |
| 3 | 1.11-2.00 | 0.99 [0.88, 1.12] | 0.898 | 0.99 [0.88, 1.12] | 0.904 | 0.99 [0.88, 1.12] | 0.904 |
| 4 | 2.00-2.09 | 1.03 [0.92, 1.17] | 0.581 | 1.02 [0.90, 1.15] | 0.749 | 1.02 [0.90, 1.15] | 0.749 |
| 5 | 2.09-2.18 | 1.01 [0.89, 1.15] | 0.882 | 0.98 [0.86, 1.12] | 0.802 | 0.98 [0.86, 1.12] | 0.803 |
| 6 | 2.18-3.00 | 1.08 [0.96, 1.22] | 0.201 | 1.07 [0.94, 1.20] | 0.303 | 1.07 [0.94, 1.20] | 0.303 |
| 7 | 3.00-3.33 | 1.09 [0.96, 1.23] | 0.168 | 1.08 [0.96, 1.22] | 0.215 | 1.08 [0.96, 1.22] | 0.215 |
| 8 | 3.33-4.44 | 1.05 [0.93, 1.19] | 0.387 | 1.04 [0.92, 1.17] | 0.550 | 1.04 [0.92, 1.17] | 0.549 |
| 9 | 4.44-6.40 | 0.99 [0.87, 1.12] | 0.822 | 0.99 [0.88, 1.12] | 0.906 | 0.99 [0.88, 1.12] | 0.907 |
| 10 | 6.40-95.80^2^ | 0.91 [0.80, 1.03] | 0.116 | 0.91 [0.80, 1.12] | 0.123 | 0.89 [0.78, 1.01] | 0.074 |

^1^ Regression models were adjusted for household crowding, winter birth, parental socioeconomic status at 8-12 years, migration status, and parental history of non-affective psychoses.

^2^ After the exclusion of individuals with ESR>22mm/h, the total range of ESR values for this decile reduced to 6.40-22mm/h.
